# Optimal Placement of Viscoelastic Vibration Dampers for Kirchhoff Plates Based on PSO Method

**DOI:** 10.3390/ma14216616

**Published:** 2021-11-03

**Authors:** Agnieszka Lenartowicz, Maciej Przychodzki, Michał Guminiak, Tomasz Garbowski

**Affiliations:** 1 Doctoral School of Poznan University of Technology, Piotrowo 3, 60-965 Poznan, Poland; agnieszka.z.lenartowicz@doctorate.put.poznan.pl; 2 Institute of Structural Analysis, Poznan University of Technology, Piotrowo 5, 60-965 Poznan, Poland; maciej.przychodzki@put.poznan.pl (M.P.); michal.guminiak@put.poznan.pl (M.G.); 3 Department of Biosystems Engineering, Poznan University of Life Sciences, Wojska Polskiego 50, 60-627 Poznań, Poland

**Keywords:** particle swarm optimization, finite element method, viscoelastic vibration dampers, thin plates, non-dimensional damping ratio, continuation method, optimal placement of dampers

## Abstract

The main subject of this study is to determine the optimal position of a fixed number of viscoelastic dampers on the surface of a thin (Kirchhoff-Love) plate. It is assumed that the dampers are described according to the generalized Maxwell model. In order to determine the optimal position of the dampers, a metaheuristic optimization method is used, called the particle swarm optimization method. The non-dimensional damping ratio of the first mode of the plate vibrations is assumed as an objective function in the task. The dynamic characteristics of the plate with dampers are determined by solving the non-linear eigenproblem using the continuation method. The finite element method is used to determine the stiffness matrix and the mass matrix occurring in the considered eigenproblem. The results of exemplary numerical calculations are also presented, where the final optimal arrangement of dampers on the surface of sample plates with different boundary conditions is shown graphically.

## 1. Introduction

The protection of engineering structure elements against the adverse effects of vibrations is an important issue in their design. Effective vibration damping is a key issue in the safety of all engineering structures, from the simplest and smallest elements of machine structures and through to machine foundations and skyscrapers. For this purpose, additional vibration damping elements are often used. Such elements include the viscoelastic vibration dampers mentioned in this paper. In many cases, the number of vibration dampers in the structure must be limited and their location cannot be random. Therefore an optimal arrangement of such dampers is often one of the most important issues to be solved. The main goal of the presented research is the optimal placement of a finite number of viscoelastic dampers inside the thin plate area. The problem of the optimal placement of dampers in building structures is a fairly frequent subject of research, but usually these studies relate to frame models—most often shear frames. The description of these studies can be found, among others in the following papers [1,2,3,4,5,6,7,8,9,10,11].

For this purpose (In order to find the best position of the dampers), a metaheuristic algorithm of particle swarm optimization might be used. Hereinafter, this will be referred to as the PSO method. The PSO method has a wide practical application in solving optimization problems. It is the subject of many scientific works in various fields. This algorithm was created in 1995 as a result of work carried out by Kennedy and Eberhart [12]. It is a stochastic calculation algorithm based on the observation of the behavior of a moving flock of birds. This algorithm makes it possible to find an approximate solution for various optimization tasks. The essence of the PSO method and its application for structure optimization tasks is described in [13], where the authors examine the influence of the algorithm coefficients on the solution of the truss optimization problem. The performance of the PSO algorithm is known to be sensitive to the values assigned to its control parameters. The results of the research on the impact of these parameters on the effectiveness of the PSO method are presented in papers [14,15,16]. In addition, a number of modifications to this optimization method were developed. Their description is in the papers [17,18,19,20,21,22]. A consistent presentation of the development path and the current state of knowledge about the PSO method can be found in the publication [23]. However, in this work the classic variant of the PSO method [13] was used.

The free damped vibrations of rectangular thin plates equipped with viscoelastic vibration dampers are analyzed in this research. Such plates might be a part of a larger structure, e.g., a part of a foundation system or protective structure. The analysis was carried out in order to find the optimal distribution of the set number of dampers on the surface of the plate in order to suppress the first mode of vibration of the plate as much as possible. For this purpose, the aforementioned PSO optimization method was used. A generalized Maxwell model described i.a. in [24,25] is used to describe the dampers. The finite element method was used to calculate the dynamic characteristics of the plate with viscoelastic dampers. The FEM model for the plate element was developed based on the well-known books [26,27] as well as [28].

In the performed numerical analyzes, it was necessary to solve the nonlinear eigenproblem. For this purpose, the continuation method presented in the paper [29] was used. This method was previously used only for multilayered beams [30,31] and 2D shear frame structures with viscoelastic dampers [29,31]. Here it was expanded and adapted to Kirchhoff’s plate elements. All calculations were performed in the Octave software through the authors implementation of the entire problem. The obtained results prove the effectiveness of the proposed approach.

The main motivation to undertake the research described here was the need to solve a practical engineering problem using relatively simple implementations. In the design practice, optimal solutions are sought that will allow the achievement of the assumed final result. In the studies under consideration, it is the effect of the maximum vibration damping of the plate element included in the engineering structure. On the basis of the obtained results, it can be concluded that both the robustness and effectiveness of the presented approach has been proven.

## 2. Theoretical Background

### 2.1. Description of Plate Model according to Finite Element Method

**Figure 1:**
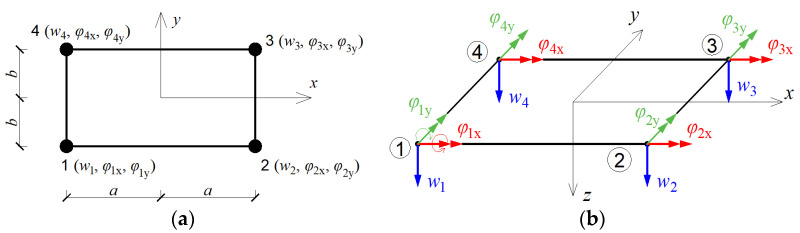
Finite element type plQ4 used for discretization of tested plates. (**a**) Node numbering, (**b**) active degrees of freedom.

In the Finite Element Method, the center plane of the plate is divided into a finite number of elements. In this study, rectangular plate finite elements of plQ4 type, described in [26,27,28] and shown in Figure 1, are used. Each such finite element is characterized by four nodes with three degrees of freedom at each node.

Thus, the deformation vector $w_{i}^{e}$ of the i-th node in the finite element e is defined by three quantities: deflection $w_{i}$ and two angles of rotation $\varphi_{ix}$ and $\varphi_{iy}$, so it can be written that:

(1) $$w_{i}^{e}={\left[\begin{array}{ccc} w_{i} & \varphi_{ix} & \varphi_{iy} \end{array}\right]}^{T}={\left[\begin{array}{ccc} w_{i} & \frac{\partial w_{i}}{\partial y} & -\frac{\partial w_{i}}{\partial x} \end{array}\right]}^{T};i=1,2,3,4.$$

The displacement field within each finite element is approximated with a fourth order polynomial $w^{e}\left(x,y\right)$ of two variables x and y, the formula of which is given in [28]. This polynomial has twelve unknown coefficients $\alpha_{k}$ (k=1,2,3,…,12) due to the number of degrees of freedom in one finite element.

For each finite element e, twelve shape functions $N_{k}^{e}\left(x,y\right)$ (k=1,2,3,…,12) are determined. Each shape function $N_{k}^{e}\left(x,y\right)$ corresponds to the k-th degree of freedom of the element and is determined based on the displacement field $w^{e}\left(x,y\right)$ formula. For this, an appropriate system of twelve equations is solved. Knowing all the shape functions of an element, the displacement field within the element e can be expressed as a linear combination of the shape functions $N_{k}^{e}\left(x,y\right)$, with coefficients being nodal displacements, i.e.,

(2) $$w^{e}\left(x,y\right)=N_{e}w_{e},$$

where $w_{e}={\left[w_{1}^{e},\;w_{2}^{e},\;w_{3}^{e},\;w_{4}^{e}\right]}^{T}$ and $N_{e}=\left[N_{1}^{e},\;N_{2}^{e},\;N_{3}^{e},\;\ldots N_{12}^{e}\right]$.

On the basis of the knowledge of the shape functions, it is possible to determine the element stiffness matrix $K_{e}$ and the consistent inertia matrix $M_{e}$ of the element. The formulas for determining these matrices are given in [26,27,28]. The dimension of the stiffness and inertia matrices $K_{e}$ and $M_{e}$ is 12×12 due to the twelve degrees of freedom of a single finite element.

### 2.2. The Viscoelastic Damper Model

In this paper, a description of viscoelastic damper according to a generalized Maxwell model is assumed. This model is discussed, inter alia, in [24,25].

**Figure 2:**
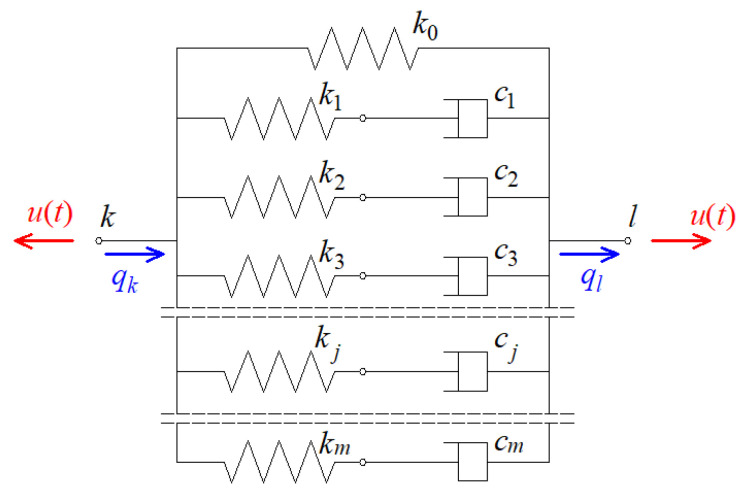
Generalized Maxwell model of viscoelastic damper.

Using the classic description, the viscoelastic damper can be shown graphically as in Figure 2. It can be seen from the figure that the damper consists of m elements called Maxwell elements and an additional spring element. Each of the Maxwell elements contains a viscous part with the constant $c_{j}$ (dashpot) and an elastic part (spring) with the constant $k_{j}$ where j=1,2,…,m.

The time-dependent force in the damper, denoted as u(t), is the sum of the forces occurring in the individual elements, i.e.,

(3) $$u\left(t\right)=\overset{m}{\underset{j=0}{\sum }}u_{j}\left(t\right).$$

For j=0, the force in the spring element is expressed by the following formula:

(4) $$u_{0}\left(t\right)=k_{0}\Delta q\left(t\right),$$

where $k_{0}$ is the stiffness parameter of the spring element and $\Delta q\left(t\right)=q_{l}\left(t\right)-q_{k}\left(t\right)$ is the relative displacement of the damper (i.e., the difference of the displacements of the ends l and k of the damper). For j=1,2,…,m, the force in the j-th Maxwell element satisfies the following formula:

(5) $$\nu_{j}u_{j}\left(t\right)+{\dot{u}}_{j}\left(t\right)=k_{j}\Delta \dot{q}\left(t\right)$$

where $\nu_{j}=k_{j}/c_{j}$ is the quotient of the stiffness and damping coefficients of the j-th Maxwell element.

Using Laplace transform (ℒ-transform) with zero initial conditions for formulas (4) and (5) causes them to transform into forms:

(6) $${\bar{u}}_{0}\left(s\right)=k_{0}\Delta \bar{q}\left(s\right)$$

(7) $${\bar{u}}_{j}\left(s\right)=\frac{k_{j}s}{\nu_{j}+s}\Delta \bar{q}\left(s\right)$$

In the above formulas, s is an ℒ-transform variable and ${\bar{u}}_{j}\left(s\right)$, $\Delta \bar{q}\left(s\right)$ are respectively ℒ-transforms of the time-dependent force function $u_{j}\left(t\right)$ in the j-th damper element and the relative displacement function Δq(t) of the damper. Laplace transform of the total force u(t) in the damper takes the following form:

(8) $$\bar{u}\left(s\right)=\overset{m}{\underset{j=0}{\sum }}{\bar{u}}_{j}\left(s\right)=\left(k_{0}+\overset{m}{\underset{j=1}{\sum }}\frac{k_{j}s}{\nu_{j}+s}\right)\Delta \bar{q}\left(s\right).$$

Formula (8) can be re-written in a shorter form as below:

(9) $$\bar{u}\left(s\right)=\left(K_{r}+G_{r}\left(s\right)\right)\Delta \bar{q}\left(s\right),$$

where

(10) $$K_{r}=k_{0};\;G_{r}\left(s\right)=\overset{m}{\underset{j=1}{\sum }}\frac{k_{j}s}{\nu_{j}+s}.$$

### 2.3. The Equation of Motion of the Plate with VE Dampers and Solution of Eigenproblem

The equation of motion of a structure with viscoelastic dampers can be written in the following form:

(11) $$M\ddot{q}\left(t\right)+C\dot{q}\left(t\right)+Kq\left(t\right)=f\left(t\right).$$

In the above equation, K, M and C denote the global plate stiffness, inertia and damping matrices, respectively. The dimension of these matrices is 3n×3n, where n is the total number of nodes of all finite elements making up the plate. There are also two vectors in the equation: q(t) is the 3n-dimensional plate nodal displacement vector and f(t) is the vector of the forces acting on the plate from dampers. It is assumed in the equation that the structural plate is not loaded with additional excitation forces.

The matrices K and M appearing in Equation (11) arise as a result of the aggregation process of element matrices $K_{e}$ and $M_{e}$ respectively. In numerical tests included in this paper, the damping matrix C is adopted as the proportional damping matrix, i.e., it is a linear combination of the matrices **K** and **M**.

After applying the Laplace transform with zero initial conditions, Equation (11) takes the following algebraic form:

(12) $$\left(s^{2}M+sC+K\right)\bar{q}\left(s\right)=\bar{f}\left(s\right),$$

where $\bar{q}\left(s\right)$ is the ℒ-transform of q(t) and $\bar{f}\left(s\right)$ can be expressed as follows:

(13) $$\bar{f}\left(s\right)=-\overset{n_{d}}{\underset{r=1}{\sum }}\left(K_{r}+G_{r}\left(s\right)\right)L_{r}\bar{q}\left(s\right).$$

In the formula above, $n_{d}$ is the total number of dampers attached to the plate at selected nodes of a finite element mesh and $L_{r}$ is a global matrix indicating the location of the r-th damper within the plate. It is a diagonal matrix with one in the row representing the translational degree of freedom along which the r-th damper works. Formulas for determining $K_{r}$ and $G_{r}\left(s\right)$ for the r-th damper are given in Section 2.2.

After substituting (13) to (12), the ℒ-transform of the Equation (11) of motion of a plate with viscoelastic dampers takes the form:

(14) $$\left(s^{2}M+sC+K+K_{d}+G_{d}\left(s\right)\right)\bar{q}\left(s\right)=0,$$

where

(15) $$K_{d}=\overset{n_{d}}{\underset{r=1}{\sum }}K_{r}L_{r},\;$$

(16) $$G_{d}\left(s\right)=\overset{n_{d}}{\underset{r=1}{\sum }}G_{r}\left(s\right)L_{r}.$$

Equation (14) is a nonlinear eigenproblem which is solved by eigenvalues s and eigenvectors $\bar{q}\left(s\right)$. This problem can be solved e.g., according to the algorithm of the continuation method described in more detail in [29] and used in this paper.

In the case of Equation (14), the components containing the variable s in the first power are multiplied by the parameter κ∈[0;1]. The equation can then be written as

(17) $$h_{1}\left(\bar{q},s\right)=D\left(s\right)\bar{q}\left(s\right)=0,$$

where

(18) $$D\left(s\right)=s^{2}M+\kappa sC+K+K_{d}+\kappa G_{d}\left(s\right).$$

In order for the elements of the eigenvector $\bar{q}$ corresponding to the eigenvalue s to be determined unambiguously, an additional normalizing equation of the following form is introduced into the matrix Equation (17):

(19) $$h_{2}\left(\bar{q},s\right)=\frac{1}{2}\bar{q}{\left(s\right)}^{T}\frac{\partial D\left(s\right)}{\partial s}\bar{q}\left(s\right)-a=0,$$

where a has a given value.

In the first step of the continuation method, in Equation (16) the parameter $\kappa_{1}=0$ is assumed and the generalized eigenproblem is solved. As a result of solving this problem, the first approximations of eigenvalues $s_{1}^{\left(1\right)},s_{2}^{\left(1\right)},\ldots ,s_{3n}^{\left(1\right)}$ and eigenvectors ${\bar{q}}_{1}^{\left(1\right)},{\bar{q}}_{2}^{\left(1\right)},\ldots ,{\bar{q}}_{3n}^{\left(1\right)}$ are obtained. On their basis, the parameter $a_{j}^{\left(1\right)}=s_{j}^{\left(1\right)}{\left({\bar{q}}_{j}^{\left(1\right)}\right)}^{T}M{\bar{q}}_{j}^{\left(1\right)}$, where j=1,2,…,3n, is determined.

In the l-th step (l=2, 3, 4,…) the increment $\Delta \kappa_{l}$ is assumed and the Newton method is used to solve the system of Equation (17) with the additional Equation (19). For this purpose, the system of incremental equations of the Newton method is solved using $\kappa_{l}=\kappa_{l-1}+\Delta \kappa_{l}$, $s_{j}^{\left(k-1\right)}$, ${\bar{q}}_{j}^{\left(k-1\right)}$ and $a_{j}^{\left(k-1\right)}$. Increments $\delta \bar{q}$ and δs are obtained from these equations and the following are calculated:

(20) $$s_{j}^{\left(k\right)}=s_{j}^{\left(k-1\right)}+\delta s,$$

(21) $$\;{\bar{q}}_{j}^{\left(k\right)}={\bar{q}}_{j}^{\left(k-1\right)}+\delta \bar{q},$$

(22) $$a_{j}^{\left(k\right)}=\frac{1}{2}{\left({\bar{q}}_{j}^{\left(k\right)}\right)}^{T}\frac{\partial D\left(s\right)}{\partial s}{\bar{q}}_{j}^{\left(k\right)}.$$

Successive approximations of the j-th eigenvalue and j-th eigenvector in the l-th step of the algorithm are calculated until the desired accuracy of the final result is achieved.

The final values of $s_{j}^{\left(k\right)}$, ${\bar{q}}_{j}^{\left(k\right)}$ and $a_{j}^{\left(k\right)}$ obtained in the l-th step are taken as starting values for the step l+1 and the new parameter $\kappa_{l+1}=\kappa_{l}+\Delta \kappa_{l+1}$.

The procedure described above is carried out up to the value of the parameter κ=1, when the final eigenvalues and eigenvectors for the nonlinear eigenproblem (14) are obtained.

The obtained eigenvalues of the problem (14) are complex numbers of the form $s_{j}=\mu_{j}+i\eta_{j}$. On this basis, the j-th natural frequency $\omega_{j}$ of the structure and the non-dimensional damping ratio $\gamma_{j}$ of the j-th mode of vibration are determined from the formulas:

(23) $$\omega_{j}^{2}=\mu_{j}^{2}+\eta_{j}^{2};\;\gamma_{j}=-\frac{\mu_{j}}{\omega_{j}}.$$

In the research, the optimal placement of the vibration dampers in the plate area is sought. In this case the optimal placement of the dampers is understood to mean their position for which the first form of vibration will be suppressed to the maximum. Such a situation occurs when the non-dimensional damping ratio takes its maximum value. The continuation method makes it possible to calculate the non-dimensional damping ratio for a single mode of vibration without having to solve the entire eigenproblem.

### 2.4. Fundamentals of Particle Swarm Method

The aim of this paper is the selection of the optimal location of viscoelastic dampers supporting the Kirchhoff plate. The particle swarm method (PSO) was used to solve this problem. It is one of the gradientless optimization methods. Its algorithm is based on searching the space of possible solutions by the so-called particles [12]. Particles can be interpreted as points moving in a multidimensional space. The particle coordinates are the current values of the variables of the considered optimization process. The number of particles in a given task is constant, and their set is called a swarm. The first step of the PSO algorithm is to determine the initial position of the particles by randomly selecting points from the solution space. Each of these points corresponds to one particle. During the steps of the optimization procedure, the particles move in search of a better position, i.e., a solution for which the value of the objective function will be greater (if the maximum of the objective function is sought). Moving the swarm in the solution space is treated as a process taking place in a contractual time. The subsequent steps of the optimization procedure are treated as moments of the aforementioned contractual time. Each particle has assigned so-called neighbors, which are selected swarm particles. This assignment is usually static, meaning it takes place once and is done at the beginning of the computation.

The position of the *i*-th particle in the (*k +* 1)-th time step is given by the formula:

(24) $$x_{k+1}^{i}=x_{k}^{i}+v_{k+1}^{i}\Delta t,$$

where Δt is the time step. It is usually taken to be equal to 1. The symbol $v_{k+1}^{i}$ denotes the updated particle velocity vector, which is calculated from the relationship:

(25) $$v_{k+1}^{i}=w_{k}v_{k}^{i}+c_{1}L_{1k}\frac{p_{k}^{i}-x_{k}^{i}}{\Delta t}+c_{2}L_{2k}\frac{p_{k}^{s}-x_{k}^{i}}{\Delta t},$$

where $p_{k}^{i}$ and $p_{k}^{s}$ are the best position of the *i*-th particle and the best position of the particle in its neighborhood after *k* time steps, respectively. The symbols $L_{1k}$ and $L_{2k}$ denote diagonal matrices whose elements are random numbers with an even distribution from the interval (0,1). These numbers change with each step of the optimization procedure. The quantities $c_{1}$ and $c_{2}$ are fixed weight multipliers called cognitive and social parameters, respectively. The coefficient $w_{k}$ should be interpreted as a measure of the inertia of the particle’s motion. A broad discussion of the principles of selecting the $c_{1}$, $c_{2}$ and $w_{k}$ coefficients and their impact on the efficiency of the particle swarm method can be found in the literature [12,13,14,15,16].

In the research to which this paper is devoted, the objective function was assumed in the form of a dimensionless damping ratio of the plate. The task of the PSO algorithm is to find such a position of vibration dampers for which this ratio will assume the maximum value. It was not necessary to introduce restrictions for the objective function. The design variables are the coordinates of the position of the dampers. It was assumed that the dampers can be located in the FEM mesh nodes of the plate, which considerably simplifies the mathematical model of the analyzed structure. For the design variables, a limitation resulting directly from the model geometry was adopted—the coordinates of the damper mounting points cannot extend beyond the outer edges of the plate. The second limitation consisted in excluding the possibility of locating more than one vibration damper in the same node.

Two criteria have been defined to stop the optimization process. The first criterion concerned changes in the value of the objective function in the subsequent time steps of the procedure. It was assumed that if the best position of the whole swarm particle in the next five steps does not change, then the maximum of the objective function has been reached. The second criterion related to the number of steps in the optimization procedure. It was assumed that the number of steps should not exceed a predetermined value.

In summary, the algorithm of the particle swarm method can be written as the following steps:

1. Adopting initial positions and initial velocities of particles;
2. Checking the limiting condition of termination;
3. Calculation of the value of the objective function for the positions of individual particles;
4. Updating the best position of each particle and the best position of the particle in the vicinity of each particle after *k* iterations;
5. Determination of new positions and velocities of particles according to Formulas (24) and (25);
6. Repeating steps 2–5 until the accepted criteria for stopping the calculations are met.

## 3. Numerical Examples

**Figure 3:**
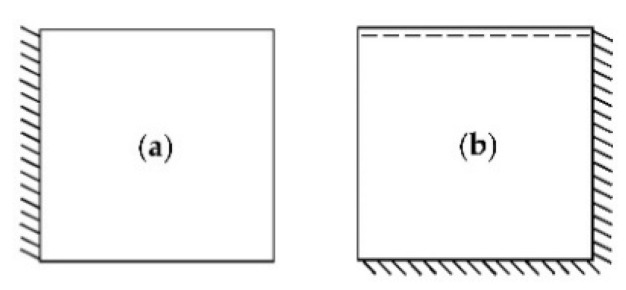
Ways of supporting the analyzed plates: (**a**) Plate clamped on one edge; (**b**) Plate clamped on two adjacent edges and simply-supported on the third edge.

This section presents the results of PSO optimization carried out on the example of an isotropic rectangular plate. Plates with two different means of support are considered. The first plate considered is clamped on one edge. In turn, the second plate under consideration is clamped on two adjacent edges and simply-supported on the third edge. Both ways of supporting the plates are shown in Figure 3. The dimensions of both plates are $l_{x}\times l_{y}\times h=\left(2.2\times 2.2\times 0.02\right)$ m. The adopted material properties of plates are as follows: $E=205\;\mathrm{GPa},\;\nu =0.3,\;\rho =7850\;{\mathrm{kg}/m}^{3}$.

The optimization task consists in arranging a fixed number of dampers on the surface of the plate. It is assumed that the viscoelastic dampers are attached to the plate with one end in points of its surface and the other end to the rigid base. All dampers are the same, each containing one spring element and one Maxwell element. Due to the fact that the aim of the research is to determine the optimal position of the dampers in the area of the plate, and not to test the effectiveness of the dampers themselves, the selection of the parameters of the damper model plays a rather secondary role. In order that the values of the damper parameters do not differ significantly from the parameters of the actual models of viscoelastic dampers, they were adopted using the data available in the literature [32] and adjusted to the given problem. The values adopted in this way and assumed for the reference temperature $T_{0}$ = 0.2 °C are as follows:

(26) $$k_{0}=108.56\;N/m;\;k_{1}=19968.09\;N/m;\;c_{1}=229.63\;\mathrm{Ns}/m.\;$$

The following assumptions are made in the optimization task under consideration using the PSO method:

- Plate discretization with FEM mesh with dimensions of 10×10 or 15×15;
- The assumed objective function—non-dimensional damping ratio of the first mode of vibration;
- Possible locations of the viscoelastic dampers—internal nodes of the FEM mesh discretizing the plate;
- The method of determining the optimal position of the assumed number of dampers—obtaining the maximum value of the objective function;
- The initial velocities of the swarm particles in each environment are assumed to be zero;
- Accepted values of cognitive parameter and social parameter: $c_{1}=c_{2}=1.0$;
- Accepted value of the inertia weight: w=0.75;
- The adopted maximum number of iterations of the PSO algorithm: 15.

### 3.1. Example No 1

In this subsection, a plate fixed on one edge, as shown in Figure 3a, is considered. It is assumed that one damper should be located on the surface of the plate so that the damping of the first mode of vibrations is as high as possible. With this assumption, the value of the non-dimensional damping ratio γ for the first mode of vibration should be maximized.

In this example, two neighborhood cases for swarm particles will be considered:

1. One neighborhood is assumed within the plate and four potential positions of swarm particles are randomly selected;
2. Four neighborhoods are assumed within the plate and four potential positions of swarm particles in each neighborhood are randomly selected

**Figure 4:**
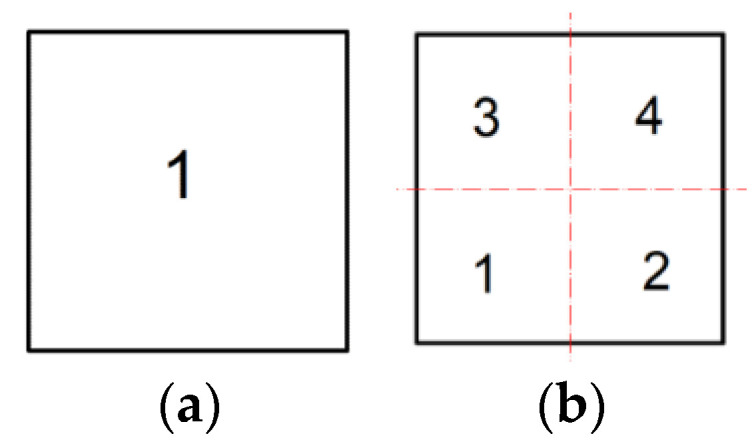
The adopted neighborhoods in the plate for swarm particles: (**a**) One neighborhood on the entire surface of the plate; (**b**) Four neighborhoods for the individual quarters of the plate.

In case 1, the neighborhood covers the entire surface of the plate. However, in case 2, individual neighborhoods are assumed in the subsequent quarters of the plate surface, as shown in Figure 4. Since one damper is to be arranged on the surface of the plate, a swarm particle is identified with a single damper and characterized by *x* and *y* coordinates defining its position on the plate surface. As mentioned earlier, the swarm consists of four particles randomly taken in each neighborhood isolated on the surface of the plate.

**Figure 5:**
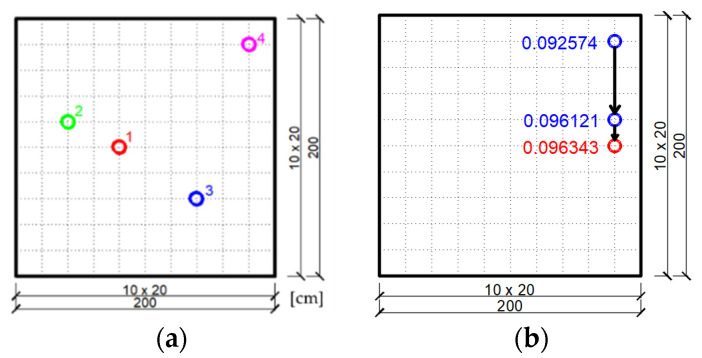
Optimization result of the plate fixed on the left edge, assuming one neighborhood on its surface: (**a**) Initial swarm of particles (individual dampers) chosen randomly for the plate; (**b**) The most favorable positions of the swarm particles achieved in subsequent iterations of the PSO algorithm.

In the considered example, discretization of the plate surface is adopted using a FEM mesh with dimensions of 10×10. Possible positions of the dampers are assumed in the inner nodes of the mesh formed in this way. Figure 5a shows the initial, random selection of swarm particles (dampers) for the case of a task with one neighborhood on the surface of the plate. In this initial swarm, particle number 4 takes the most favorable position because the non-dimensional damping ratio for this location is the largest. This is the most favorable position of the swarm particle in the zero iteration. Figure 5b shows how the best location of the particles of a four-element swarm changes in subsequent iterations of the PSO algorithm execution. At the individual most favorable positions of the particles, the corresponding value of the objective function, which is the non-dimensional damping ratio, is given. The best position of the damper on the plate surface is marked in red in Figure 5b and the value of the objective function γ=0.096343 corresponds to it.

**Figure 6:**
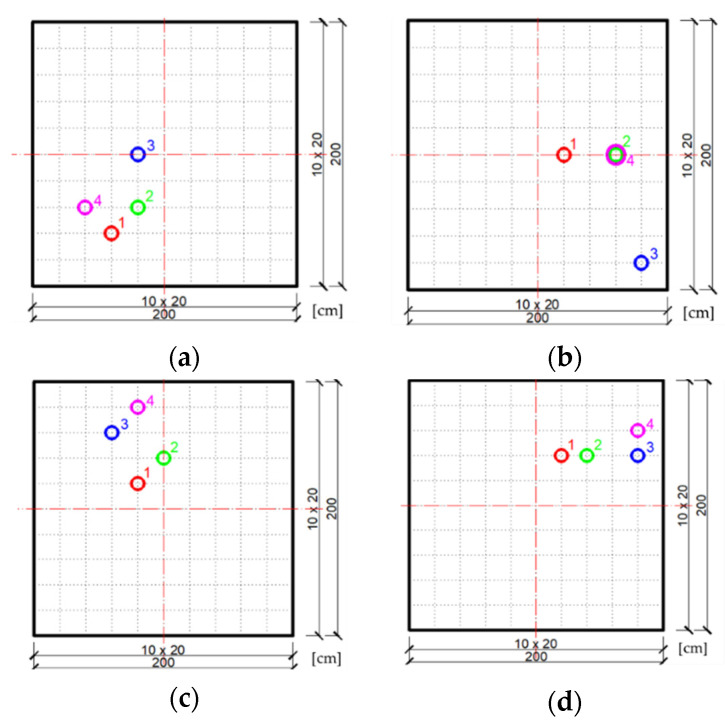
Initial swarm of particles (individual dampers) chosen randomly in individual plate neighborhoods: (**a**) Neighborhood 1; (**b**) Neighborhood 2; (**c**) Neighborhood 3; (**d**) Neighborhood 4.

Further, the task of finding the optimal location of the damper will be solved for the case of four neighborhoods in the area of the plate, as shown in Figure 4b. The randomly chosen positions of four swarm particles in each of the neighborhoods are shown in Figure 6a–d.

**Figure 7:**
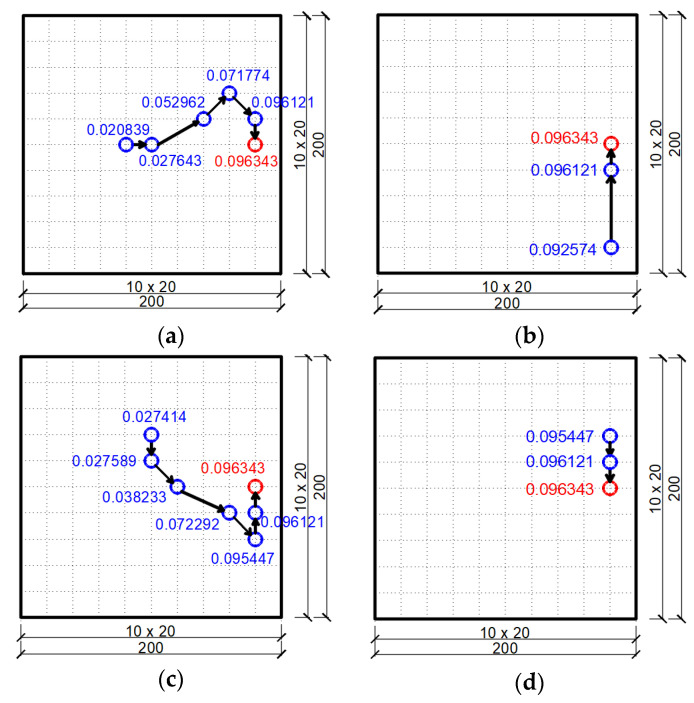
The most favorable positions of the swarm particles achieved in subsequent iterations of the PSO algorithm in individual plate neighborhoods: (**a**) Neighborhood 1; (**b**) Neighborhood 2; (**c**) Neighborhood 3; (**d**) Neighborhood 4.

Figure 7a–d shows the most favorable positions of the swarm particles in subsequent iterations of the PSO algorithm for individual neighborhoods. It can be seen that regardless of the choice of neighborhoods, the optimal final position of the damper occurs near the center of the free edge of the plate, as it was in the previous case of a plate with one neighborhood. This means that the assumed objective function has no local extremes on the plate surface and the most advantageous particle moves to the global extremum from each of its neighborhoods.

From the above-discussed cases of one or more assumed neighborhoods on the surface of the plate, it follows that the optimal location of one viscoelastic damper occurs near the center of the free edge of the cantilevered plate.

### 3.2. Example No 2

In this subsection, a plate fixed on two adjacent edges and simply supported on the third edge, as shown in Figure 3b, is considered. The reason for assuming such conditions for supporting the plate was the willingness to adopt unusual boundary conditions and create an asymmetric problem in which the optimal placement of the dampers is not obvious in advance. It is assumed that four dampers should be located on the surface of the plate. As in example 1, the damping of the first mode of vibrations should be as high as possible. With this assumption, the value of the non-dimensional damping ratio γ for the first mode of vibration should be maximized.

In the present example, two neighborhoods are assumed on the surface of the plate and eight potential positions of the four dampers in each neighborhood. Contrary to the previous example, the exact division of the plate surface into the neighborhoods is not assumed now. Swarm particles from each neighborhood are taken completely randomly.

**Figure 8:**
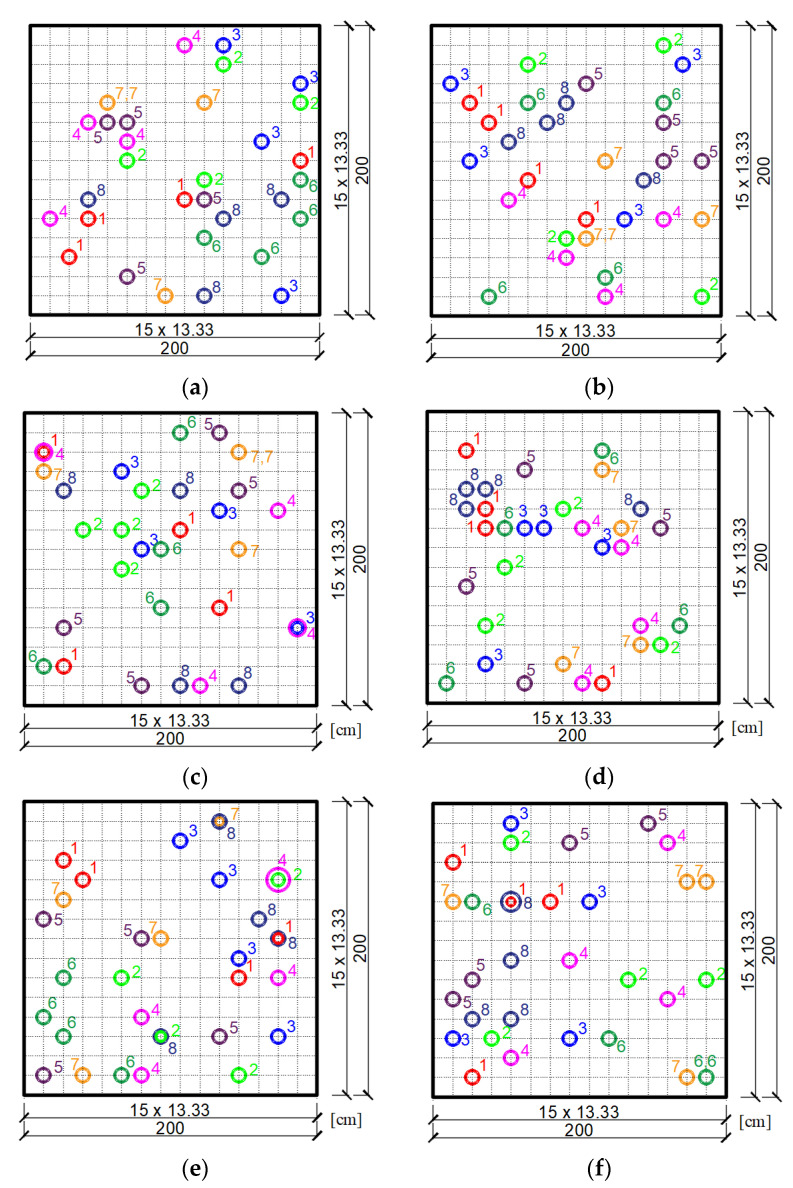
Initial swarm of particles (groups of four dampers) chosen randomly for the plate: (**a**) Neighborhood 1 for draw 1; (**b**) Neighborhood 2 for draw 1; (**c**) Neighborhood 1 for draw 2; (**d**) Neighborhood 2 for draw 2; (**e**) Neighborhood 1 for draw 3; (**f**) Neighborhood 2 for draw 3.

It should be noted that now a particle of the swarm is a set of four dampers, not a single damper as it was before. Thus, each swarm particle is characterized by eight coordinates (the *x* and *y* coordinates of individual dampers that make up a four-element swarm particle). Figure 8 shows randomly adopted swarm particles (i.e., groups of four dampers) for each of the two plate neighborhoods for three cases of draw. Individual groups of four dampers are marked in the figure with successive numbers from 1 to 8. Discretization of the plate was adopted using a FEM mesh with dimensions of 15×15.

**Figure 9:**
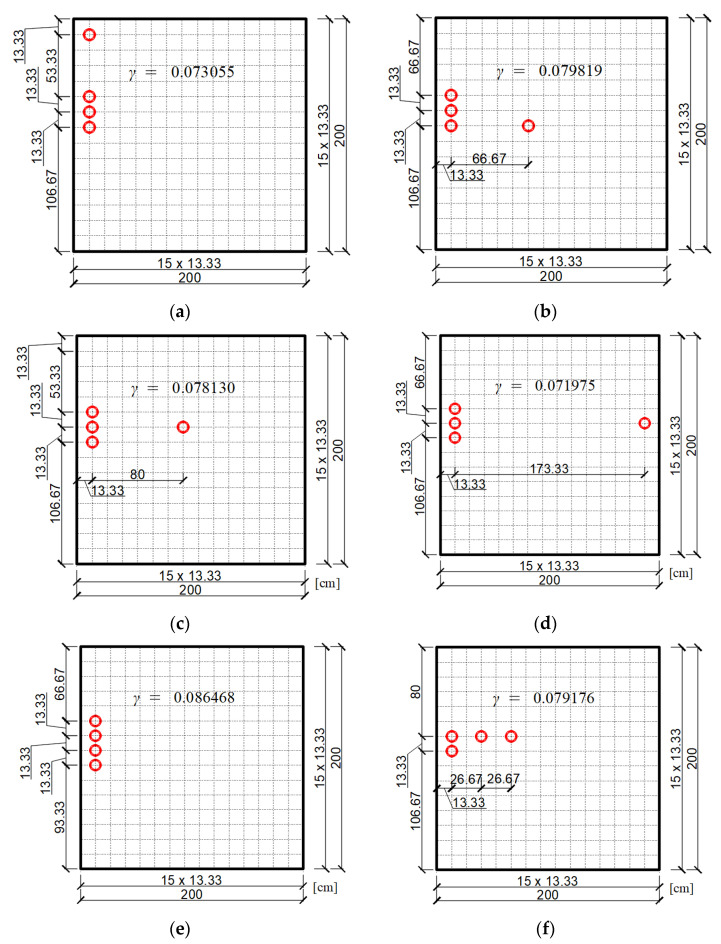
Optimal position of a group of four dampers according to the PSO algorithm for two initially adopted neighborhoods: (**a**) Neighborhood 1 for draw 1; (**b**) Neighborhood 2 for draw 1; (**c**) Neighborhood 1 for draw 2; (**d**) Neighborhood 2 for draw 2; (**e**) Neighborhood 1 for draw 3; (**f**) Neighborhood 2 for draw 3.

The final result of the optimization using the PSO method is shown in Figure 9, starting with the preliminary selection of a swarm according to the neighborhoods in Figure 8. The figure also shows the values of the objective function for each final position of the dampers.

**Figure 10:**
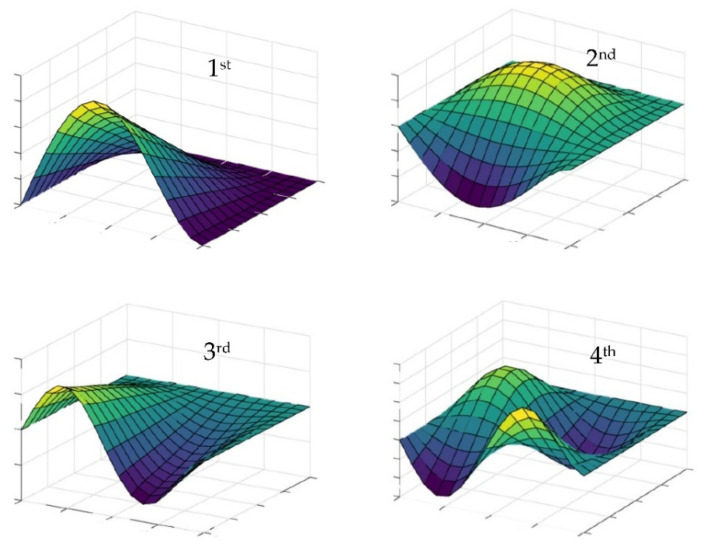
Diagrams of the first four modes of undamped vibrations for the plate mounted according to Figure 3b.

Based on the optimization results obtained from the two neighborhoods for three cases of draw, it can be concluded that they differ slightly from each other. This may result from the fact that in the swarm method only a close to optimal solution is sought. The initial selection of the position of the swarm particles may have some influence on the accuracy of the final result. Hence, in more complex tasks, where more than one damper is selected, it is more advantageous to assume several neighborhoods initially and make several draws of their placement. Then, as the optimal position, the one that corresponds to the greater value of the non-dimensional damping ratio should be chosen. In the considered example, the one shown in Figure 9e was adopted as the final optimal location of the dampers. To some extent, this location can be predicted on the basis of the graph of the first mode of undamped vibrations of the plate, which is shown in Figure 10. This figure shows the first four modes of undamped vibrations. As can be seen, the arrangement of the three dampers repeated for most of the cases from Figure 9 is at the extreme point of the graph of the first mode of undamped vibration from Figure 10.

In order to determine the influence of the optimal position of the dampers on the dynamic characteristics of the tested plate, the first four values of the natural frequency were determined. The obtained results are summarized in Table 1. The case of a plate without dampers for different discretization of the plate surface with FEM mesh and the case of a plate with dampers for their position according to Figure 9e was taken into account.

## 4. Discussion

In this paper, the method of optimal selection of viscoelastic vibration dampers on the surface of the plate was presented based on the particle swarm optimization algorithm. The dampers were selected to obtain the greatest possible damping of the first mode of vibration by maximizing the corresponding function of the non-dimensional damping ratio.

The selection of cognitive and social parameters as well as the weight of inertia has a large impact on the final results of optimization using the swarm method. The selection of cognitive and social parameters, i.e., $c_{1}$,and $c_{2}$ respectively, shows how the speed of the current particle depends on itself or on the entire swarm. In the considered problem of the optimal selection of dampers, the adoption of the same values of both coefficients, i.e., $c_{1}=c_{2}=1.0$, turned out to be the most advantageous. Taking one of these factors over the other resulted in less satisfactory final results. The weight of inertia w could be adopted from the range (0,1). The research conducted by the authors allowed for the conclusion that it is more advantageous to adopt the value of the inertia coefficient closer to 1. Then the swarm particle velocities are updated more widely and the solution tends to reach the optimal level faster and more precisely. Hence, in the considered examples, it was assumed that w=0.75.

It is worth discussing the influence of the initial selection of swarm particles on the final results. In the examples considered earlier, the initial swarm was selected randomly. In example 1, regardless of the selection of swarm particles and the number of neighborhoods, the final position of the damper was the same. On the other hand, in example 2, the final results differed for the randomly selected swarms from the two neighborhoods. Therefore, it can be concluded that for more complex problems it is more advantageous to choose more than one neighborhood and solve the same task several times for different selections of swarm particles. If the final results are similar to each other, the optimal solution should be chosen in a way that allows for the obtaining of a more favorable value of the objective function.

At the end of the discussion, it is worth mentioning the selection of the objective function. In this study, the non-dimensional damping ratio was adopted. Then, in the iterative process of the PSO method, the value of the objective function thus assumed should be calculated repeatedly. The process of calculating the non-dimensional damping ratio is also iterative, so the final results of the PSO algorithm are obtained at the expense of considerable computational time. Therefore, in order to speed up the PSO optimization algorithm, in the considered tasks of optimizing the position of dampers in plates it would be necessary to test for the selection of another objective function.

## 5. Conclusions

The PSO method applied to the optimal selection of viscoelastic dampers on the plate surface presented in this paper allows for the obtaining of satisfactory results. The method allows for the finding of a solution close to the optimal one. The final result of the task is significantly influenced by the selection of the PSO coefficients and the initial adoption of the set of swarm particles. It is advisable to select several environments in order to compare the quality of final results. On the other hand, the use of the continuation method to solve the nonlinear eigenproblem in Kirchhoff plates turned out to be a very promising approach which not only allows for a quick calculation of the plate vibration eigenvalues, but is also very precise. So far, in the vast majority of works, the continuation method has been presented for structures with a one-dimensional description of deformation i.e., beams and shear frames.

The adoption of the optimal position of the dampers resulted in an increase in the frequency of natural vibrations for the first mode of vibrations, which is the most important in the design of dynamically loaded structures. In order to achieve a more significant change in the dynamic characteristics of the plate, it would be necessary to adopt a larger number of dampers and/or change their parameters. Taking into account the presence of viscoelastic dampers, an increase in the natural frequency of the structure-dampers system is additionally obtained. The viscoelastic damper contains a stiffness element, and thus the resultant stiffness of the entire system becomes greater. This results in an increase in the frequency of free circular vibrations of the plate-damper system compared to the pure structure (plate). A more pronounced effect of the presence of dampers will be visible for forced vibrations analysis (harmonically or not), which will show a decrease in the amplitudes of displacements in selected points in the structure, i.e., in finite element nodes. Another element motivating us to take up this topic is to demonstrate the usefulness of the continuation method.

The presented method can be improved by changing the objective function, which can significantly improve the computational burden compared to the one based on the dimensionless damping factor that was considered in this preliminary study. All calculations were carried out using the authors’ implementation in the Octave symbolic programming language, therefore each adoption and improvement can be done efficiently. The presented solution can be also easily transferred to the dynamics of the structural system consisting, for example, of a plate (foundation) resting on a finite number of vibro-isolators which do not have to be symmetrically or regularly arranged.

## Figures and Tables

**Table 1:** The natural frequencies of the plate from Example 2 disregarding the influence of the presence of dampers and taking them into account at the optimal location obtained.

Natural Frequencies ω [rad/s]			
Mode	Plate without Dampers with Different FEM Discretization		Plate with Dampers Located according to Figure 9e
	Mesh 10 × 10	Mesh 15 × 15	
1	67.808	67.802	72.896
2	138.279	138.812	141.802
3	200.620	200.449	201.151
4	271.529	273.269	273.679

## Data Availability

The data presented in this study are available on request from the corresponding author.
